# 

**Published:** 1888-02

**Authors:** 


					﻿rolutne EVI.]
FEBRUARY, 1888;
5
[Number 2/
M^f"gWWMK1
HEsH B A W
■Mmikkfaw
o w[<£l fil 11JI ^1 if
EDITOR:
S. J. JONES, M.D., LL.D
Rand, McNally & Company, Printers.
1888.
Entered at the Post Office at Chicago, for transmission through the mails as seeend-olaas matter.
				

